# Biomechanical analysis of skeletal open bite treatment methods: a comparative finite element study

**DOI:** 10.55730/1300-0144.6191

**Published:** 2026-01-19

**Authors:** Ömer Faruk SARI, Muhammed Hilmi BÜYÜKÇAVUŞ

**Affiliations:** 1 Department of Orthodontics, Faculty of Dentistry, Ankara Medipol University, Ankara, Turkiye; 2 Department of Orthodontics, Faculty of Dentistry, Antalya Bilim University, Antalya, Turkiye

**Keywords:** Open bite, finite element analysis, intrusion, zygomatic plate, headgear

## Abstract

**Background/aim:**

The biomechanical effects of zygomatic anchorage plates, vertical chin-caps, and occipital headgear on dentofacial structures in patients with skeletal anterior open bite malocclusion were compared using three-dimensional finite element analysis.

**Materials and methods:**

Three-dimensional finite element models were constructed based on computed tomography data obtained from a patient with skeletal anterior open bite. Simulation models representing zygomatic anchorage-supported intrusion, vertical chin-cap, and occipital headgear were generated under fixed orthodontic conditions. In each scenario, a unilateral force of 200 g (1.96 N) was applied along clinically relevant force vectors. Stress distributions in craniofacial bones, sutural structures, teeth, roots, and temporomandibular joint components, as well as displacement patterns of the teeth and roots, were evaluated. Von Mises stress was used to assess craniofacial and dental structures, whereas minimum principal stress was used to evaluate sutural regions.

**Results:**

The highest von Mises stress within craniofacial structures was observed in the posterior maxilla in the zygomatic anchorage plate scenario, followed by the vertical chin-cap and occipital headgear scenarios. Across all models, compressive stresses in sutural structures were predominantly concentrated in the zygomaticomaxillary suture, with the greatest magnitude observed in the zygomatic anchorage model. Dental and root stress analyses revealed peak von Mises stress at the mesiobuccal root of the maxillary first molar, particularly in the zygomatic anchorage scenario. The greatest tooth and root displacements along the vertical axis, indicative of intrusion, were also observed in this model. Condylar stress was primarily localized in the superior region of the condyle, with higher peak values observed in the vertical chin-cap simulation.

**Conclusion:**

Within the limitations of finite element analysis, zygomatic anchorage-supported intrusion demonstrated greater predicted posterior maxillary intrusion and higher localized stress concentrations than vertical chin-cap and occipital headgear approaches. These findings indicate distinct biomechanical profiles among the appliances, which should be considered when selecting treatment strategies for skeletal anterior open bite.

## 1. Introduction

Anterior open bite malocclusion is considered a significant clinical challenge in orthodontics, owing to its complex etiology, prolonged treatment duration, and high propensity for relapse. Unlike many malocclusions that can be attributed to a single cause, anterior open bite typically arises from a combination of factors, necessitating a comprehensive understanding of its multifactorial etiology [1]. Historically, orthopedic appliances such as occipital headgear, posterior bite blocks, posterior magnets, functional appliances, and vertical chin-caps have been considered the mainstay of treatment for anterior open bite [2].

Cephalometric analyses have demonstrated the efficacy of the vertical chin-cap in modulating mandibular growth by promoting posterior–inferior rotation, achieving anterior–superior displacement, reducing anterior facial height, and intruding the lower posterior dentoalveolar region [3]. Similarly, occipital headgear has been used to control maxillary vertical growth and posterior tooth eruption, as well as to facilitate dental intrusion [4]. However, the introduction of skeletal anchorage systems, including miniscrews, miniplates, and miniimplants, has substantially transformed orthodontic practice. These patient-compliance-independent modalities provide robust anchorage, thereby enabling efficient execution of complex tooth movements and facilitating the treatment of challenging malocclusions. Numerous studies have reported the successful application of skeletal anchorage systems in the treatment of anterior open bite in adult patients [5–7].

Despite valuable clinical insights obtained from patient-based studies, inherent variability in growth and development necessitates rigorous control of factors influencing orthopedic treatment outcomes. To mitigate these challenges and achieve greater precision, in vitro analyses utilizing three-dimensional (3D) models are increasingly employed. Among these techniques, finite element analysis (FEA) has emerged as a powerful tool for simulating and quantifying biomechanical forces. FEA translates complex phenomenasuch as stress, strain, bending, heat transfer, and fluid flow—into solvable mathematical models, thereby providing valuable insights into orthodontic treatment mechanics [8].

The biomechanical effects of zygomatic anchorage plates, vertical chin-caps, and occipital headgear, in conjunction with posterior bite blocks and fixed mechanics, on dentofacial structures in individuals with skeletal anterior open bite malocclusion were investigated in this study. By utilizing finite element analysis, a methodology that complements 3D digital imaging techniques, the forces generated by these orthopedic appliances and their implications for treatment efficacy were elucidated.

## 2. Materials and methods

Ethical approval for this study was obtained from the Ethics Committee of Süleyman Demirel University, Isparta, Türkiye (date: 04 September 2020; approval no: 2020/243). Three-dimensional computed tomography images of a 17-year old male patient with skeletal anterior open bite malocclusion were obtained, and a simulation model was created using the analysis software. The finite element analyses were conducted using AyTasarım (AyTasarım Mühendislik Ltd. Şti., Ankara, Türkiye), a commercial finite element analysis software based on the ANSYS Mechanical APDL platform (ANSYS Inc., Canonsburg, PA, USA).

A workstation equipped with an Intel Xeon processor (Intel Corporation, Santa Clara, CA, USA) operating at 3.30 GHz, a 500 GB hard disk, 14 GB RAM, and Windows 7 Ultimate (Microsoft Corporation, Redmond, WA, USA) was used for 3D mesh organization, homogenization, solid model generation, and finite element procedures. An Activity 880 optic scanner (smart optics Sensortechnik GmbH, Bochum, Germany), Rhinoceros 4.0 3D modeling software (Robert McNeel and Associates, Seattle, WA, USA), VRMesh Studio (VirtualGrid Inc., Bellevue, WA, USA), and Algor Fempro (ALGOR Inc., Pittsburgh, PA, USA) were utilized.

All models were assumed to consist of linear, homogeneous, and isotropic materials. During mesh constructing, second-order 10-node elements, which allow higher-resolution analyses, were used whenever possible. Lower-order elements were used when necessary to complete the structure in regions close to the center of the models. Efforts were made to generate the highest possible mesh quality by maximizing nodal density to facilitate computational accuracy. The Young’s modulus values assigned in the finite element models were as follows: 18,600 MPa (megapascals) for teeth, which were modeled as a single homogeneous enamel–dentin complex; 7 MPa for the periodontal ligament; 7800 MPa for cancellous bone; 14,000 MPa for cortical bone; 7 MPa for sutural structures; 200,000 MPa for brackets and wires; 105,000 MPa for miniscrews and miniplates; 44,100 MPa for the temporomandibular joint (TMJ) disc; and 2490 MPa for polymethylmethacrylate (PMMA). The Poisson’s ratios assigned were 0.31 for teeth, 0.49 for the periodontal ligament, 0.30 for cancellous bone, 0.30 for cortical bone, 0.49 for sutural structures, 0.29 for brackets and wires, 0.33 for miniscrews, 0.40 for the TMJ disc, and 0.35 for PMMA. The total number of nodes was 222,275 in scenario 1, 206,938 in scenario 2, and 203,987 in scenario 3. The total number of elements was 919,509 in scenario 1, 876,744 in scenario 2, and 796,865 in scenario 3 (Table 1) [9].

All materials were assumed to exhibit linear, homogeneous, and isotropic behavior. The periodontal ligament (PDL) was modeled as a uniform layer of constant thickness surrounding the tooth roots, in accordance with commonly accepted finite element modeling approaches in orthodontics. The tooth–PDL and PDL–alveolar bone interfaces were defined as bonded contacts to simulate physiological load transfer without relative sliding.

In all scenarios, the dental system was modeled under fixed orthodontic conditions, including brackets and archwires. The bracket–archwire interaction was modeled as a bonded contact, assuming no relative movement between the archwire and bracket slots, to focus on the global biomechanical effects of the applied orthopedic forces rather than detailed sliding mechanics. The acrylic bite block used in the zygomatic anchorage plate and vertical chin-cap scenarios was defined using surface-to-surface contact with the occlusal surfaces of the teeth, thereby allowing force transmission while preserving physiological tooth mobility.

In the occipital headgear scenario, the inner bow (facebow) was directly connected to the tubes of the maxillary first molars, through which the applied force was transmitted. In the zygomatic anchorage plate scenario, the force was applied to the nonosseous hook portion of the plate and transmitted to the dentoalveolar structures via the acrylic plate. In the vertical chin-cap scenario, the force was applied from the inferior border of the mandible toward the occipital region, passing obliquely through the condylar axis.

The temporomandibular joint (TMJ) was modeled by defining frictionless, surface-to-surface contact between the mandibular condyle, articular disc, and glenoid fossa. This approach allowed physiological load transfer while avoiding artificial constraints that could affect stress distribution in the condylar region. To ensure the reliability of the finite element model, a basic mesh convergence assessment was performed. Mesh density was progressively refined in biomechanically critical regions, including the posterior maxilla, maxillary molar roots, sutural regions, and the temporomandibular joint. Key output parameters, including peak stress values in the posterior maxilla, the mesiobuccal root of the maxillary first molar, and the condylar region, as well as maximum tooth displacement, were monitored during mesh refinement. No relevant changes in the distribution patterns or peak magnitudes of these parameters were observed beyond the final mesh configuration, indicating mesh-independent results within the scope of this study.

In anatomically complex or biomechanically less critical regions, fewer nodal elements were selectively used to preserve mesh continuity. This approach was intended to enhance computational efficiency without compromising model integrity. In contrast, regions requiring high precision were meshed with the highest possible density to maintain the overall quality and fidelity of the finite element model.

**Figure 1:**
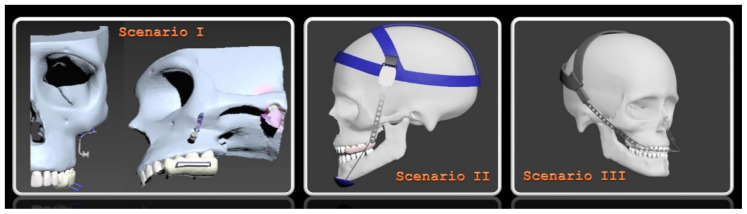
Craniofacial models illustrating the intrusion appliance with zygomatic plate anchorage, the vertical chin-cap combined with a bite block, and the occipital headgear combined with fixed mechanics.

The screws of the zygomatic plate were positioned in the model at the level of the maxillary first molar, corresponding to the inferior aspect of the zygomaticomaxillary buttress region. The nonosseous part of the plate was designed in a hook-like configuration to allow force application. In this scenario, the force was applied to the nonosseous (hook) portion of the zygomatic plate, positioned in the zygomaticomaxillary buttress region, and directed posteroinferiorly (downward and backward). The force vector was modeled to induce intrusion of the maxillary posterior teeth and was transmitted to the dentoalveolar structures via an intraoral acrylic plate (Figure 1).

The vertical chin-cap appliance was supported by the inferior region of the chin and the occipital region of the head, with anchorage provided at the occipital side and force transmission influenced by the mandibular border. The appliance components corresponding to these regions were modeled as two distinct sections. The connecting component was positioned obliquely to approximate the trajectory passing through the condylar axis. The vertical chin-cap system was modeled to provide support from the inferior border of the mandible toward the occipital region of the head. The force was directed in a posterosuperior direction, obliquely upward from the chin, and passing over the condylar axis. The connecting component was placed on an inclined plane within the model to realistically reflect clinical application (Figure 1).

The intraoral facebow component of the occipital headgear appliance was designed according to the material properties of steel wire, while the extraoral component was positioned to form an angle of approximately 45° with the occlusal plane. The components supported by the facebow and head region were modeled in combination with the intermediate component. In this model, the inner bow (facebow) was directly attached to the tubes of the maxillary first molars. The force was applied in a posterosuperior direction at an angle of approximately 45°, consistent with clinical practice (Figure 1).

In this study, the dental system in all three scenarios was modeled under fixed orthodontic conditions, including brackets and archwires. Specifically, in the occipital headgear scenario, the inner bow (facebow) component was directly connected to the tubes of the maxillary first molars, through which force transmission was achieved. In the zygomatic plate and vertical chin-cap scenarios, the intraoral acrylic plate was positioned in contact with the teeth; however, no direct fixation or splinting was applied. Therefore, during the definition of contact elements in the model, a contact approach was adopted to reflect physiological tooth mobility while ensuring surface interaction among the tooth–archwire/bracket–periodontal ligament–alveolar bone interfaces.

A unilateral force of 200 g (1.96 N) was applied in all three scenarios. In this study, stress values were expressed in megapascals (MPa = N/mm^2^), and displacement values were expressed in millimeters (mm). The von Mises stress values presented in this study represent the peak stress levels observed in the relevant regions of each structure, including bone, suture, tooth, and root. These values were extracted from nodal regions in which the highest stress concentrations were identified by the FEA software. In the evaluation of displacement magnitudes, the X-axis represented the transverse plane, the Y-axis represented the sagittal plane, and the Z-axis represented the vertical plane.

### 2.1. Boundary conditions

Boundary conditions were defined to provide a stable reference framework while allowing realistic load transfer through the craniofacial structures. The mandible was constrained at its posterior and inferior regions by fixing all degrees of freedom, a commonly used approach in finite element studies to simulate cranial support and prevent rigid body motion. This assumption allowed the applied orthopedic forces to be transmitted through the dentofacial structures without introducing numerical instability.

In all three scenarios, a force magnitude of 200 g (1.96 N) was applied to isolate the biomechanical effects of appliance design rather than differences in applied force magnitude. Although higher force levels are frequently used in clinical practice for vertical chin-cap and occipital headgear applications, the same force magnitude was selected across all models to enable direct comparison of stress distributions and displacement patterns among the appliances.

In the zygomatic anchorage plate scenario, the force was applied to the nonosseous hook portion of the plate located in the zygomaticomaxillary buttress region and was directed posteroinferiorly toward the maxillary posterior dentition via the acrylic bite block. In the vertical chin-cap scenario, the force was applied from the inferior border of the mandible and transmitted in a posterosuperior direction, passing obliquely through the condylar axis toward the occipital region. In the occipital headgear scenario, the force was applied through the inner bow connected to the maxillary first molar tubes, with the extraoral component oriented at approximately 45° to the occlusal plane, consistent with clinical application.

These boundary condition and loading assumptions were selected to reflect clinically relevant force vectors while maintaining consistency across scenarios, thereby allowing meaningful biomechanical comparison of the three treatment modalities.

Because the values obtained from finite element modeling are derived from deterministic algebraic calculations without variance, statistical analyses cannot be performed. At this stage, emphasis was placed on the precise evaluation and interpretation of cross-sectional images, as well as the magnitude and distribution of stress at the nodal level.

Frictionless, surface-to-surface contact was defined for the temporomandibular joint components and selected interfaces where relative motion was required, whereas bonded contact was used for the tooth–PDL, PDL–bone, and bracket–archwire interfaces to ensure stable load transfer. This contact definition was applied to the interface between the mandibular condyle and the glenoid fossa, opposing dental surfaces, the tooth–periodontal ligament interface, and the periodontal ligament–alveolar bone interface.

## 2. Results

The stress distributions in craniofacial structures, sutural structures, teeth, and roots are presented in Table 2. Von Mises stress was used to evaluate stress distribution in craniofacial bones, teeth, and roots, whereas minimum principal stress was used for sutural structures to better represent compressive loading patterns. Accordingly, negative values indicate compressive stresses within the sutural regions. The displacements of teeth and roots are shown comparatively for all three scenarios in Table 3. In the tables, the X-axis represents the buccolingual direction, the Y-axis represents the mesiodistal direction, and the Z-axis represents the vertical direction. Positive values indicate buccal tipping along the X-axis, mesialization along the Y-axis, and intrusion along the Z-axis, whereas negative values indicate palatal tipping along the X-axis, distalization along the Y-axis, and extrusion along the Z-axis.

**Figure 2:**
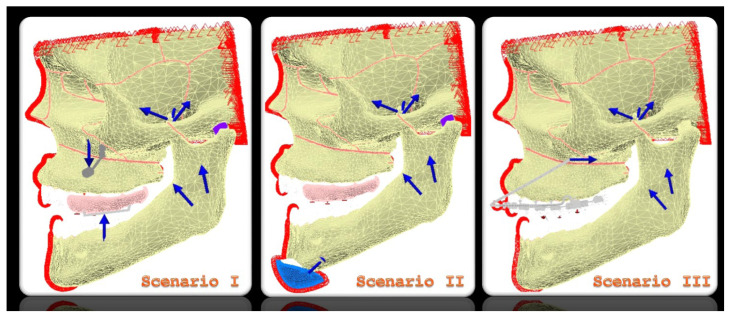
Illustration of force application points on the model. Blue arrows indicate the points and directions of force application.

**Figure 3:**
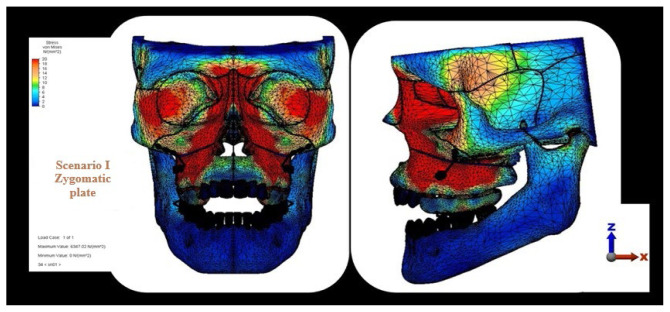
Von Mises stress distribution in craniofacial structures in the zygomatic anchorage scenario.

**Figure 4:**
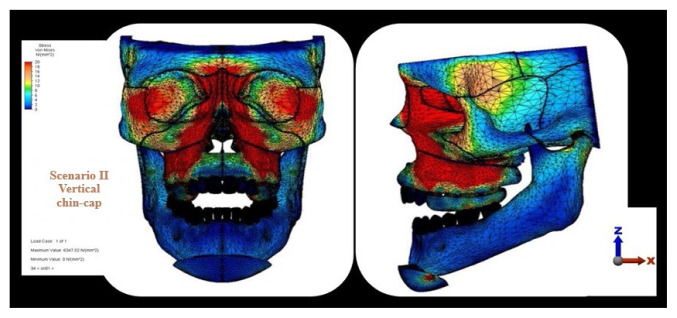
Von Mises stress distribution in craniofacial structures in the vertical chin-cap scenario.

**Figure 5:**
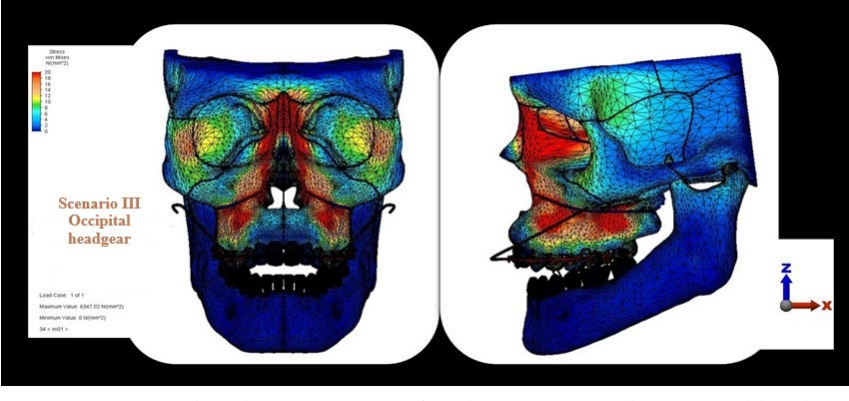
Von Mises stress distribution in craniofacial structures in the occipital headgear scenario.

The highest stress observed in the craniofacial structures, particularly within the maxilla, was localized in the posterior region of the maxillary bone; among the scenarios, this value was highest in the zygomatic anchorage plate scenario and lowest in the occipital headgear scenario. In the occlusal view, the zygomatic plate anchorage scenario exhibited the highest stress values, which were primarily detected on the first premolar (Figures 2–5).

**Figure 6:**
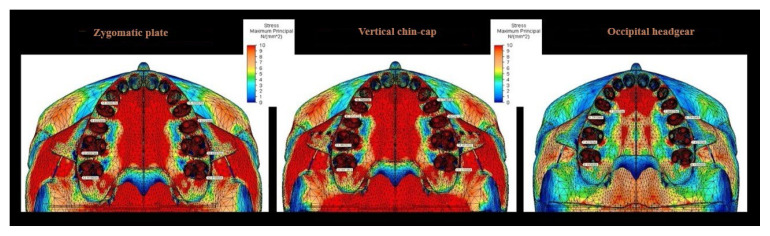
Minimum principal stress distribution in sutural structures across all three scenarios. Negative values represent compressive stresses.

Von Mises stress values in the sutural structures were evaluated across five regions, with the highest values observed in the zygomaticomaxillary suture, where intrusive movement was most pronounced. The highest stress levels in this region were detected in the zygomatic anchorage, vertical chin-cap, and occipital headgear scenarios, respectively (Figure 6).

**Figure 7:**
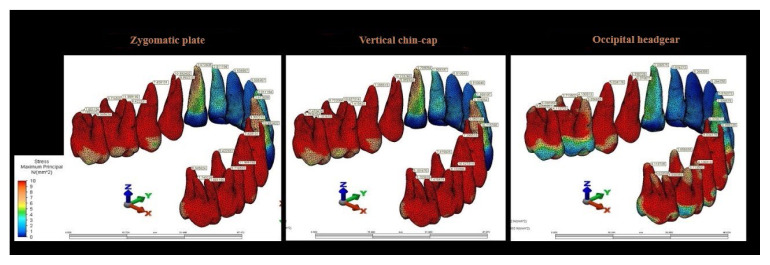
Von Mises stress distribution in tooth roots across all scenarios.

Evaluation of stress in the tooth roots revealed that the highest von Mises stress values occurred in the zygomatic anchorage plate scenario and were localized at the mesiobuccal root of the maxillary first molar (Figure 7).

**Figure 8:**
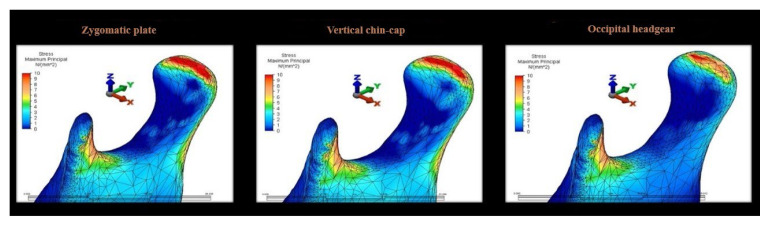
Maximum principal stress distribution in the mandibular condyle across all three scenarios.

Total displacement magnitudes of teeth and tooth roots were also evaluated separately along the X-, Y-, and Z-axes. In the zygomatic anchorage plate scenario, the highest displacement along the X-axis was observed at the mesiobuccal root of the first molar (0.29 mm). Similarly, in the occipital headgear scenario, the mesiobuccal root of the first molar exhibited the greatest displacement. When total tooth displacement was considered, the highest value was again observed at the first molar (0.034 mm) in both the zygomatic anchorage plate and occipital headgear scenarios (Table 3). Along the Y-axis, the highest displacement was observed in the zygomatic anchorage scenario, whereas in the occipital headgear scenario, the greatest displacement occurred at the distobuccal root of the second molar (−0.06724 mm). In terms of total tooth displacement, the highest values were observed in the zygomatic anchorage scenario and at the second premolar in the occipital headgear scenario (Table 3). Along the Z-axis, the highest displacement was observed at the buccal root of the first premolar (0.214 mm) in the zygomatic anchorage plate scenario, while the greatest total tooth displacement was observed at the second premolar (0.474 mm) in the same scenario (Table 3). The distribution and color scale of von Mises stress values in the condyle are shown in Figure 8. Accordingly, the highest stress values within the condylar structure were observed in the superior region of the condyle, followed by the coronoid process, across all three scenarios. The lowest stress values were observed in the coronoid notch region. Among the three appliances, the highest stress values were observed in the vertical chin-cap scenario (Figure 8). The peak maximum principal stress values and their anatomical locations within the mandibular condyle for all three scenarios are summarized in Table 4. The maximum and minimum stress and displacement values described in the Results section were cross-checked against the numerical data presented in Tables 2 and 3, and complete consistency was confirmed across the text, tables, and figure legends.

## 3. Discussion

The treatment of skeletal anterior open bite malocclusions presents a multifaceted challenge, with a range of orthopedic appliances—including occipital headgear, posterior bite blocks (both active and passive), and vertical chin-caps—often being employed in combination [10–12]. In recent years, skeletal anchorage–supported molar intrusion has emerged as a viable alternative to orthognathic surgery, with studies reporting outcomes comparable to those of surgical interventions [13].

Erverdi et al. demonstrated the efficacy of miniplates placed on the zygomatic buttress for molar intrusion in the correction of open bite, using an intraoral appliance with acrylic plates and transpalatal arches [14]. In the present study, an acrylic bite block was incorporated into both the zygomatic anchorage plate and vertical chin-cap scenarios. However, to isolate the effects of the applied intrusion force, the influence of masticatory forces was deliberately excluded from the model.

The magnitude of force application represents a critical parameter in orthodontic treatment. Erverdi et al. applied a total of 400 g bilaterally for block molar intrusion [15], whereas Çifter and Saraç utilized 300 g per segment in their finite element analysis (FEA) of different force application points [16]. In the present study, a unilateral force of 200 g was selected for the zygomatic plate simulation, in accordance with established experimental and clinical protocols employing acrylic plate–mediated force application [15,17]. This decision, although differing from the 300 g force applied via archwires by Çifter and Saraç, ensured comparability with studies utilizing acrylic plate–mediated force application.

For the vertical chin-cap, a force of 200 g was applied and directed vertically through the condyle to ensure consistency across appliances. Similarly, for the occipital headgear, a force of 200 g was simulated, with the inner bow terminating at the molar tubes and the outer bow adjusted to generate intrusive effects, using the occipital region as the support point. Although chin-cap and headgear applications often utilize forces between 300 g and 500 g [18,19], a force of 200 g was maintained across all scenarios to isolate the impact of appliance design, rather than force magnitude, on treatment outcomes. This approach facilitated a more precise comparison of the resulting biomechanical effects.

The accuracy of FEA is contingent upon mesh quality, including node and element density. In this study, a refined mesh with increased element count and detailed anatomical representations was employed, thereby enhancing the fidelity of the simulations compared to previous FEA studies [20].

The current analysis revealed distinct stress distributions across the craniofacial structures. In the zygomatic plate and vertical chin-cap scenarios, the highest stress accumulation was observed in the posterior maxillary and dental regions. Notably, in the zygomatic plate scenario, the magnitude of stress was higher than that observed in the vertical chin-cap scenario. This finding is consistent with the results reported by Erverdi et al. [14], which demonstrated intense mechanical effects in the posterior maxilla. Although such stress levels may enhance the effectiveness of intrusion, they should be carefully evaluated with respect to the load-bearing capacity of the periodontal tissues. This finding aligns with the direct application of force to the posterior maxilla, with subsequent stress propagation to adjacent bones via sutural connections. In contrast, the occipital headgear scenario exhibited peak stress concentrations in the zygomatic bone. This pattern of stress distribution, in which regions proximal to force application exhibit higher stress levels, is consistent with established FEA principles [16]. The bite plate and zygomatic anchorage plate likely contributed to the increased stress observed in the posterior teeth and maxilla in the first two scenarios.

Evaluation of von Mises stress in the sutural structures demonstrated peak stress in the zygomaticomaxillary suture across all scenarios. In the present study, compressive stress distribution in sutural regions was evaluated using minimum principal stress values, as this metric more accurately reflects sutural biomechanics under orthopedic loading. Notably, the zygomatic anchorage model displayed the greatest stress magnitude, consistent with the findings of Çifter and Saraç [16], who observed that buccally applied intrusive forces generate maximal stress concentrations in the midfacial sutures. These findings highlight the mechanical sensitivity of the sutural interfaces to skeletal anchorage force vectors. While the occipital headgear scenario exhibited comparable stress levels in the zygomaticofrontal suture, the zygomaticomaxillary suture remained the dominant site. These findings diverge from the results reported by Alkan and Akkaya, who identified predominant stress in the frontomaxillary and nasomaxillary sutures [19]. This discrepancy may be attributed to the bite block–enhanced force system used in the zygomatic plate and vertical chin-cap scenarios, as well as variations in mesh density and geometric model complexity.

Analysis of von Mises stress and displacement in teeth and roots revealed peak stress in the maxillary posterior teeth—particularly the first premolar and second molar—in the zygomatic anchorage plate and vertical chin-cap scenarios. The lower stress observed in the vertical chin-cap and occipital headgear scenarios is likely attributable to the direct application of intrusive force through the teeth. These findings corroborate the observations of Uysal et al., who reported peak stress in the vestibular tubercles of premolars and second molars [17], and align with the findings of Alkan and Akkaya, who described stress distribution in the buccal tubercle of the maxillary second premolar and molars [19]. The higher stress observed in the molar teeth in the occipital headgear scenario may be attributed to the direct connection of the intraoral arm of the facebow to the molar band.

Root stress analysis revealed peak stress in the mesiobuccal root of the maxillary first molar, particularly in the zygomatic anchorage scenario. This root exhibited approximately twice the stress intensity compared with that observed in the occipital headgear scenario. This observation is clinically relevant, as Uysal et al. [17] previously emphasized the vulnerability of this root under intrusive loading, raising concerns regarding potential root resorption during prolonged treatment. The zygomatic plate scenario exhibited the highest root stress overall, followed by the vertical chin-cap scenario, whereas the occipital headgear scenario showed distinctly lower stress values. The discrepancy in stress distribution across scenarios may be attributed to the use of skeletal anchorage in the zygomatic plate scenario. These findings align with the report of Uysal et al., who identified peak stress in the mesiobuccal root of the first molar [17], as well as with the observations of Çifter and Saraç, who reported peak stress in the mesiobuccal root of the first molar and premolar roots [16].

Clinical studies have documented molar movement patterns consistent with these findings. Çetin observed distal molar movement with occipital headgear and mesial movement with a vertical chin-cap [21], whereas Beycan and Erverdi reported 2.5 mm of molar intrusion using miniscrew anchorage [22]. In the present study, using a force of 200 g, intrusion values of 0.37 mm with the zygomatic plate, 0.341 mm with the vertical chin-cap, and 0.235 mm with the occipital headgear were observed.

Condylar stress analysis revealed peak stress in the condylar head across all scenarios, consistent with the findings of Tanaka et al., who reported potential avascular necrosis–like changes in the condyle [23]. The observation of peak condylar stress in the vertical chin-cap scenario suggests potential temporomandibular joint implications, particularly in patients with preexisting joint conditions. The exclusion of masticatory forces from the model allowed for the isolation of appliance-induced stress.

Limitations of this study include the use of a single craniofacial model, which may not fully capture individual anatomical variability, as well as the assumption of fixed material properties for bone and soft tissues. Additionally, the exclusion of masticatory forces represents a simplification of clinical reality. Despite these limitations, the consistency of the findings with clinical observations underscores the utility of FEA as a valuable tool for biomechanical evaluation in orthodontics.

Several limitations of this study should be acknowledged. First, the finite element model was constructed using computed tomography data from a single 17-year old male patient, which limits the representation of anatomical variability and restricts the generalizability of the findings. Therefore, the results should be interpreted as hypothesis-generating biomechanical evidence rather than definitive clinical predictions. In addition, the assumptions of linear, homogeneous, and isotropic material properties, together with the exclusion of masticatory forces, represent simplifications of clinical reality. Despite these limitations, the model allowed for controlled comparison of force systems and provided valuable insight into the relative biomechanical behavior of different orthopedic appliances.

## 4. Conclusion

Direct intrusive forces applied in the zygomatic plate and vertical chin-cap scenarios resulted in peak stress concentrations within the posterior maxilla and dentition. Conversely, the occipital headgear scenario exhibited maximum stress in the zygomatic bone. The zygomaticomaxillary suture consistently demonstrated the highest stress levels, indicating its pivotal role in force dissipation. The zygomatic plate system, characterized by a high potential for posterior maxillary intrusion and associated maximal stress concentration, may represent an effective treatment option for skeletal open bite cases.

The zygomatic anchorage plate produced the largest tooth and root displacements, particularly in the mesiobuccal root of the first molar and the buccal root of the first premolar. While all appliances achieved molar intrusion, the zygomatic plate yielded the greatest magnitude, followed by the vertical chin-cap and the occipital headgear.

The occipital headgear and vertical chin-cap scenarios, characterized by lower stress profiles, may be more suitable for growth modification approaches and less invasive treatment strategies.

Consistent peak stress was observed in the condylar head across all scenarios, with the vertical chin-cap inducing the highest stress levels, suggesting potential temporomandibular joint implications. Condylar stress analysis revealed appliance-dependent differences in peak maximum principal stress values, with the highest values observed in the vertical chin-cap scenario.

In this study, the biomechanical effects of three orthopedic appliances—the zygomatic plate, vertical chin-cap, and occipital headgear—were compared in anterior open bite cases using finite element analysis. The zygomatic plate scenario produced the greatest amount of intrusion and the highest stress concentration in the posterior maxilla, indicating the most effective skeletal-level intrusive force. However, these elevated stress values, particularly around molar roots and sutural structures, warrant caution because of potential biological risks such as root resorption. In contrast, the vertical chin-cap and occipital headgear scenarios exhibited lower stress profiles and appear to represent clinically appropriate options for growth modification–focused and less invasive interventions. These findings highlight the importance of considering not only mechanical effectiveness but also individual factors, such as patient age, growth potential, and tissue tolerance, when selecting an appropriate appliance. Additionally, the evaluation of varying force magnitudes would be beneficial for future studies.

## Figures and Tables

**Table 1:** Young’s modulus and Poisson’s ratio.

Materials	Young’s modulus (MPa)	Poisson’s ratio
Teeth (enamel–dentin complex)	18,600	0.31
Periodontal ligament	7	0.49
Cancellous bone	7800	0.3
Cortical bone	14,000	0.3
Sutural structures	7	0.49
Bracket and wires (stainless steel)	200,000	0.29
Miniscrew and miniplate (titanium alloy)	105,000	0.33
TMJ Disc	44,100	0.4
Plastic materials (ABS)	2490	0.31
PMMA (Acrylic bite block)	3000	0.35
	**Total of nodes**	**Total of elements**
Scenario 1	222,275	919,509
Scenario 2	206,938	876,744
Scenario 3	203,987	796,865

**Note:** Teeth were modeled as a single homogeneous structure representing the enamel–dentin complex. All materials were assumed to be linear, homogeneous, and isotropic, in accordance with commonly accepted finite element modeling practices in orthodontics. ABS: acrylonitrile butadiene styrene.

**Table 2:** Stress values in craniofacial structures, sutural structures, teeth, and roots across all three scenarios (MPa). Von Mises stress was used for craniofacial and dental structures, and minimum principal stress for sutural structures.

	Skeletal anchorage	Occipital headgear	Vertical chin-cap
Craniofacial structures			
Frontal bone	9.47	8.05	1.66
Zygomatic bone	14.37	12.52	6.19
Posterior maxillary bone	44.81	12.8	2.99
Temporal bone	9.81	9.81	5.18
Nasal bone	13.84	11.48	4.08
Sutural structures			
Frontomaxillary suture	−0.2	−0.2	−0.21
Zygomaticofrontal suture	−0.26	−0.04	−0.03
Zygomaticomaxillary suture	−0.007	−0.006	−0.04
Apertura piriformis	−0.48	−0.42	−1.2
Nasomaxillary suture	−0.03	−0.04	−0.11
Teeth			
First premolar tooth	16.7	15.32	7.06
Second premolar tooth	10.57	9.62	6.79
First molar tooth	11.34	10.65	7.42
Second molar tooth	14.91	12.81	8.19
Roots			
Second molar tooth palatal root	8.34	8.1	4.1
Second molar tooth distobuccal root	7.74	5.75	5.62
Second molar tooth mesiobuccal root	7.8	7.74	5.03
First molar tooth palatal root	8.42	7.41	3.55
First molar tooth distobuccal root	9.71	8.73	3.71
First molar Tooth mesiobuccal root	11.9	10.62	4.13
Second premolar tooth	7.42	7.29	5.02
First premolar tooth palatal root	9.99	9.95	6.37
First premolar tooth buccal root	10.99	10.71	6.99
Canine tooth	3.67	3.7	2.33
Lateral incisor	2.0	1.95	0.87
Central incisor	0.53	0.51	0.26

**Note:** Von Mises stress values were used to evaluate stress distribution in craniofacial bones, teeth, and roots. Minimum principal stress values were used for sutural structures to represent compressive stress distribution. Negative values indicate compressive stresses within the sutural regions.

**Table 3:** Displacement magnitudes of teeth and roots across all three scenarios.

	Skeletal anchorage			Occipital headgear			Vertical chin-cap		
	X Axis	Y Axis	Z Axis	X Axis	Y Axis	Z Axis	X Axis	Y Axis	Z Axis
Teeth									
First premolar tooth	0.01	−0.162	0.428	0.0059	−0.129	0.460	−0.0952	−0.012	0.506
Second premolar tooth	−0.01	−0.144	0.474	0.014	−0.139	0.401	−0.0041	0.032	0.357
First molar tooth	0.034	−0.15	0.341	0.031	−0.132	0.312	0.0811	−0.025	0.219
Second molar tooth	0.032	−0.146	0.333	0.033	−0.105	0.345	0.0195	−0.047	0.413
Roots									
Second molar tooth palatal root	−0.003	−0.6756	0.124	−0.001	−0.0637	0.122	−0.0013	−0.036	0.073
Second molar tooth distobuccal root	−0.001	−0.06724	0.127	−0.003	−0.0619	0.119	−0.0014	−0.035	0.075
Second molar tooth mesiobuccal root	0.014	−0.0715	0.154	0.014	−0.0665	0.149	0.0083	−0.038	0.0902
First molar tooth palatal root	0.009	−0.082	0.172	0.007	−0.0755	0.157	0.0055	−0.046	0.0900
First molar tooth distobuccal root	0.022	−0.089	0.187	0.021	−0.0828	0.177	0.0132	−0.050	0.101
First molar tooth mesiobuccal root	0.029	−0.097	0.206	0.028	−0.0896	0.195	0.0154	−0.052	0.109
Second premolar tooth	0.01	−0.087	0.205	0.01	−0.0809	0.195	0.0073	−0.049	0.115
First premolar tooth palatal root	0.007	−0.09	0.199	0.0074	−0.0837	0.190	0.0053	−0.051	0.113
First premolar tooth buccal root	0.011	−0.097	0.214	0.011	−0.0904	0.206	0.0075	−0.055	0.121
Canine tooth	0.0007	−0.074	0.179	0.0007	−0.0688	0.171	0.0016	−0.045	0.103
Lateral incisor	−0.0014	−0.079	0.185	−0.0014	−0.0733	0.176	−0.0004	−0.046	0.104
Central incisor	−0.0015	−0.076	0.192	−0.0015	−0.0706	0.183	−0.0009	−0.044	0.106

**Table 4:** Peak maximum principal stress values and their anatomical locations in the mandibular condyle across all three scenarios.

Scenario	Stress metric	Peak stress value (MPa)	Location of peak stress
**Zygomatic anchorage plate**	Maximum principal stress	~8.5	Superior region of the condylar head
**Vertical chin-cap**	Maximum principal stress	~9.8	Superior region of the condylar head
**Occipital headgear**	Maximum principal stress	~5.6	Superior region of the condylar head

**Note:** Peak stress values were estimated based on the color scale and nodal peak regions illustrated in Figure 8. Maximum principal stress was used to represent compressive and tensile stress patterns in the condylar region.

## Data Availability

The datasets used and/or analyzed during the current study are available from the corresponding author upon reasonable request.

## References

[1] Subtelny JD Sakuda M Open-bite: Diagnosis and treatment American Journal of Orthodontics 1964 50 5 337 358 10.1016/0002-9416(64)90175-7

[2] Tanaka E Iwabe T Watanabe M Kato M Tanne K An adolescent case of anterior open bite with masticatory muscle dysfunction The Angle Orthodontist 2003 73 5 608 613 14580031 10.1043/0003-3219(2003)073<0608:AACOAO>2.0.CO;2

[3] Işcan HN Dinçer M Gültan A Meral O Taner-Sarisoy L Effects of vertical chincap therapy on the mandibular morphology in open-bite patients American Journal of Orthodontics Dentofacial Orthopedic 2002 122 5 506 511 10.1067/mod.2002.128643 12439479

[4] Proffit WR Contemporary Orthodontics Contemporary Orthodontics: Contemporary Orthodontics-E-Book Elsevier Health Sciences 2018

[5] Pisani L Bonaccorso L Fastuca R Spena R Lombardo L Systematic review for orthodontic and orthopedic treatments for anterior open bite in the mixed dentition Progress in Orthodontics 2016 17 1 14 10.1186/s40510-016-0142-0 27615261 PMC5027197

[6] Sherwood KH Burch JG Thompson WJ Closing anterior open bites by intruding molars with titanium miniplate anchorage American Journal of Orthodontics Dentofacial Orthopedic 2002 122 6 593 600 10.1067/mod.2002.128641 12490869

[7] Verrue V Dermaut L Verhegghe B Three-dimensional finite element modelling of a dog skull for the simulation of initial orthopaedic displacements The European Journal of Orthodontics 2001 23 5 517 527 10.1093/ejo/23.5.517 11668871

[8] Richmond BG Wright BW Grosse I Dechow PC Ross CF Finite element analysis in functional morphology In: Anatomical Record-Part A Discoveries in Molecular, Cellular, and Evolutionary Biology 2005 10.1002/ar.a.20169 15747355

[9] Sarrafpour B Rungsiyakull C Swain M Li Q Zoellner H Finite element analysis suggests functional bone strain accounts for continuous posteruptive emergence of teeth Archives of Oral Biology 2012 57 1070 1078 10.1016/j.archoralbio.2012.05.001 22673755

[10] Sankey WL Buschang PH English J Albert HO Early treatment of vertical skeletal dysplasia: The hyperdivergent phenotype American Journal of Orthodontics Dentofacial Orthopedic 2000 118 3 317 327 10.1067/mod.2000.106068 10982934

[11] Conley RS Legan HL Correction of severe vertical maxillary excess with anterior open bite and transverse maxillary deficiency The Angle Orthodontist 2002 72 3 265 274 12071611 10.1043/0003-3219(2002)072<0265:COSVME>2.0.CO;2

[12] Cozza P Mucedero M Baccetti T Franchi L Early orthodontic treatment of skeletal open-bite malocclusion: a systematic review The Angle Orthodontist 2005 75 5 707 713 16279818 10.1043/0003-3219(2005)75[707:EOTOSO]2.0.CO;2

[13] Kuroda S Sakai Y Tamamura N Deguchi T Takano-Yamamoto T Treatment of severe anterior open bite with skeletal anchorage in adults: comparison with orthognathic surgery outcomes American Journal of Orthodontics and Dentofacial Orthopedics 2007 132 5 599 605 10.1016/j.ajodo.2005.11.046 18005833

[14] Erverdi N Usumez S Solak A New generation open-bite treatment with zygomatic anchorage The Angle Orthodontist 2006 76 3 519 526 16637736 10.1043/0003-3219(2006)076[0519:NGOTWZ]2.0.CO;2

[15] Erverdi N Keles A Nanda R The use of skeletal anchorage in open bite treatment: a cephalometric evaluation The Angle Orthodontist 2004 74 3 381 390 15264651 10.1043/0003-3219(2004)074<0381:TUOSAI>2.0.CO;2

[16] Çifter M Saraç M Maxillary posterior intrusion mechanics with mini-implant anchorage evaluated with the finite element method American Journal of Orthodontics and Dentofacial Orthopedics 2011 140 5 233 241 10.1016/j.ajodo.2011.06.019 22051501

[17] Uysal C Baloş Tuncer B Tuncer C Maxillary posterior intrusion with corticotomy-assisted approaches with zygomatic anchorage-a finite element stress analysis Progress in Orthodontics 2019 20 1 1 12 10.1186/s40510-019-0262-4 30828752 PMC6397825

[18] Maruo IT Maruo H Saga AY de Oliveira DD Argenta MA Tridimensional finite element analysis of teeth movement induced by different headgear forces Progress in Orthodontics 2016 17 1 1 9 10.1186/s40510-016-0130-4 27264500 PMC4893458

[19] Alkan Ö Akkaya S An investigation of the biomechanical effects of vertical chin cup on mandibular condyle and ramus in those with and without anterior openbite: a finite element method Acta Odontologica Turcica 2020 37 2 48 53 10.17214/gaziaot.657363

[20] Rahmitasari F Ishida Y Kurahashi K Matsuda T Watanabe M PEEK with reinforced materials and modifications for dental implant applications Dentistry Journal 2017 5 4 35 10.3390/dj5040035 29563441 PMC5806965

[21] Çetin E Comprasion of the effects of posterior bite block-vertical chincap and posterior bite block-occipital headgear treatment methods on the skeletal morphology of facial and dentoalveolar structures in the early treatment of anterior skeletal open bite Phd Süleyman Demirel Üniversitesi Sağlık Bilimleri Enstitüsü Isparta, Türkiye 2009

[22] Beycan K Erverdi N Anterior open-bite treatment by means of zygomatic miniplates: a case report Journal of Istanbul University Faculty of Dentistry 2017 51 1 52 56 10.17096/jiufd.20633 28955587 PMC5573496

[23] Tanaka E Yamano E Inubushi T Kuroda S Management of acquired open bite associated with temporomandibular joint osteoarthritis using miniscrew anchorage Korean Journal of Orthodontics 2012 42 3 144 154 10.4041/kjod.2012.42.3.144 23112945 PMC3481975

